# Platycodin D Inhibits Inflammatory Response in LPS-Stimulated Primary Rat Microglia Cells through Activating LXRα–ABCA1 Signaling Pathway

**DOI:** 10.3389/fimmu.2017.01929

**Published:** 2018-01-09

**Authors:** Yunhe Fu, Zhuoyuan Xin, Bin Liu, Jiaxin Wang, Jingjing Wang, Xu Zhang, Yanan Wang, Fan Li

**Affiliations:** ^1^Department of Clinical Veterinary Medicine, College of Veterinary Medicine, Jilin University, Changchun, China; ^2^Department of Pathogenobiology, The Key Laboratory of Zoonosis, Chinese Ministry of Education, College of Basic Medicine, Jilin University, Changchun, China; ^3^Cardiovascular Disease Center, First Hospital of Jilin University, Changchun, China

**Keywords:** platycodin D, LPS, TLR4, NF-κB, LXRα

## Abstract

Platycodin D (PLD), an effective triterpenesaponin extracted from *Platycodon grandiflorum*, has been known to have anti-inflammatory effect. In the present study, we investigate the anti-inflammatory effects of PLD on LPS-induced inflammation in primary rat microglia cells. The results showed that PLD significantly inhibited LPS-induced ROS, TNF-α, IL-6, and IL-1β production in primary rat microglia cells. PLD also inhibited LPS-induced NF-κB activation. Furthermore, our results showed that PLD prevented LPS-induced TLR4 translocation into lipid rafts *via* disrupting the formation of lipid rafts by inducing cholesterol efflux. In addition, PLD could activate LXRα–ABCA1 signaling pathway which induces cholesterol efflux from cells. The inhibition of inflammatory cytokines by PLD could be reversed by SiRNA of LXRα. In conclusion, these results indicated that PLD prevented LPS-induced inflammation by activating LXRα–ABCA1 signaling pathway, which disrupted lipid rafts and prevented TLR4 translocation into lipid rafts, thereby inhibiting LPS-induced inflammatory response.

## Introduction

Neuroinflammation plays a critical role in the pathogenesis of neurodegenerative diseases (1, 2). Neuroinflammation is induced by activated microglia (3). Microglia activation often occurs in response to inducers, such as bacterial pathogens (4). LPS, the outer membrane component of Gram-negative bacteria, is a potent stimulus for microglia activation (5). LPS leads to the activation of TLR4 signaling pathway, as well as the activation of NF-κB and inflammatory cytokines release in microglia (6). Overproduction of these cytokines leads to the pathogenesis of neurodegenerative diseases (7). Therefore, inhibition of these inflammatory cytokines may attenuate the development of neurodegenerative diseases. LXRα, a member of nuclear receptor superfamily, could induce the activation of ABCA1. Previous studies showed that activation of LXRα–ABCA1 signaling pathway could disrupt the formation of lipid rafts through decreasing the levels of cholesterol in lipid rafts. Furthermore, disruption of lipid rafts could inhibit TLR4 signaling pathway through preventing TLR4 translocation into lipid rafts.

Platycodin D (PLD), an effective triterpenesaponin isolated from the root of *Platycodon grandiflorum*, has been reported to have anti-inflammatory, antitumor, and antioxidative effects (8–10). PLD inhibited LPS-induced NO and TNF-α production in RAW264.7 cells (11). PLD inhibited LPS-induced acute lung injury in mice (12, 13). Furthermore, PLD protected alcohol-induced liver injury in mice (14). In addition, PLD was found to protect alloxan-induced diabetic mice *via* regulation of Treg/Th17 balance (15). PLD also had protective effects against OVA-induced allergic asthma in mice (16). The purpose of this article was to investigate the effects of PLD on LPS-stimulated inflammation in primary rat microglia cells *in vitro*. PLD significantly inhibited LPS-induced inflammatory response in microglia cells. PLD may be used as a therapeutic agent for neurodegenerative diseases.

## Materials and Methods

### Materials

Platycodin D (purity >99%) was purchased from National Institutes for Food and Drug Control (Beijing, China). LPS (*Escherichia coli* O55:B5) and MTT were purchased from Sigma (St. Louis, MO, USA). TNF-α, IL-6, and IL-1β ELISA kits were purchased from Biolegend (CA, USA). Rabbit anti-human TLR4, NF-κB p65, IκBα, and β-actin antibodies were purchased from Cell Signaling Technology (Danvers, MA, USA). Rabbit anti-human LXRα and ABCA1 antibodies were obtained from Santa Cruz Biotechnology Inc. (Santa Cruz, CA, USA).

### Cell Culture

Primary rat microglia cells were cultured as reported elsewhere (17). In brief, whole brains of 1-day-old neonatal Wistar rats were dissociated into individual cells that were cultured for 11 or 14 days as mixed glial cultures in DMEM with 10% fetal calf serum. All animal experiments were approved by the NIH Guide for the Care and Use of Laboratory Animals. The experiments were approved by the Institutional Animal Care and Use Committee of Jilin University.

### MTT Assay

Primary rat microglia cells were seeded in a 96-well plate (1 × 10^4^cells/well). Then, the cells were incubated with PLD and stimulated by LPS for 24 h. After that, MTT (5 mg/ml, 20 μl) was added to each well and incubated for 4 h. Absorbance was determined at 540 nm.

### Cytokine Assays

Primary rat microglia cells were incubated with PLD for 12 h and then stimulated by LPS for 24 h. The levels of inflammatory cytokines TNF-α, IL-6, and IL-1β in the culture supernatants were determined by ELISA kits (Biolegend, CA, USA) according to the manufacturer’s protocol.

### ROS Assay

Intracellular ROS was measured by using DCFDA-cellular reactive oxygen species detection assay kit (Abcam, Cambridge, UK) and a colorimetric assay kit specific for H_2_O_2_ (Sigma, USA) according to the manufacture’s protocol.

### Western Blot Analysis

Total proteins from primary rat microglia cells were extracted by M-PER Mammalian Protein Extraction Reagent (Thermo). The proteins were separated by 12% SDS-PAGE gel. Then the proteins were transferred onto PVDF membranes, blocked, and probed with primary antibodies for LXRα, ABCA1, TLR4, NF-κB p65, IκBα, and β-actin. Subsequently, the membranes were probed with secondary antibodies. The immunobands were visualized with enhanced-chemiluminescence western blot detection kits. The intensity was measured using Image J software.

### Isolation of Lipid Rafts and Quantification of Cholesterol Levels in Lipid Rafts

Primary rat microglia cells (1 × 10^8^ cells) were lysed in ice with 0.5% Brij in TNE buffer for 1 h. Then the lysates were mixed with equal amount of 80% sucrose in TNE buffer and overlaid with 30 and 50% sucrose in the same buffer. Samples were ultracentrifuged at 100,000 *g* at 4°C for 18 h and fractionated into 12 subfractions. Cholesterol level of lipid raft was assayed by gas–liquid chromatography as previously described (18).

### Cholesterol Replenishment Experiment

Primary rat microglia cells were incubated with PLD (5, 10, 20 µM) at 37°C for 12 h. Subsequently, the cells were incubated with water-soluble cholesterol (84 µg/mL) for 30 min and stimulated with LPS. The effects of PLD on LPS-induced cytokine production were detected as mentioned above.

### LXR Receptor Gene Assay

Primary rat microglia cells were cotransfected with β-galactosidase control vector and a luciferase reporter plasmid of LXRα using FuGENE HD transfection reagent (Roche Applied Science, Indianapolis, IN, USA). Six hours after transfection, cells were treated with PLD for 12 h. Luciferase activity was normalized by β-galactosidase activity.

### LXRα siRNA Transfections

Primary rat microglia cells were transfected with LXRα siRNA (100 nM) or control siRNA (100 nM) using using FuGENE HD transfection reagent (Roche, USA). 36 h later, the cells were treated with PLD and LPS. 24 h later, the levels of TNF-α, IL-6, and IL-1β were detected.

### Statistical Analysis

All data are expressed as means ± SD. Differences between different groups were analyzed by one-way ANOVA followed by Tukey’s multiple comparison test. *p* < 0.05 was taken as statistically significant.

## Results

### Effects of PLD on Cell Viability

**Figure 1:**
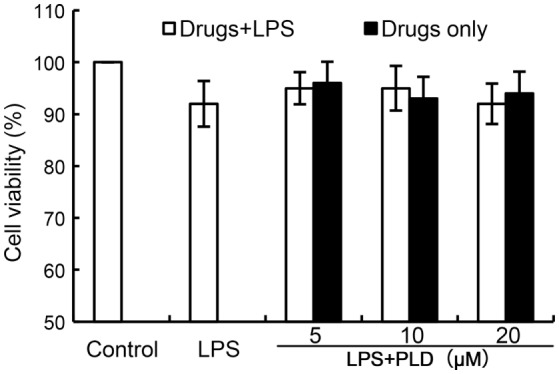
Effects of platycodin D (PLD) on the cell viability of primary rat microglia cells. Cells were cultured with different concentrations of PLD (5, 10, 20 µM) in the absence or presence of 0.5 µg/mL LPS for 24 h. The cell viability was determined by MTT assay. The values presented are the means ± SEM of three independent experiments.

The effects of PLD on the cytotoxicity of primary rat microglia cells by using an MTT assay. PLD at the doses of 0–20 µM did not affect the cell viabilities of primary rat microglia cells (Figure 1). Therefore, in the subsequent experiments, PLD were used at the doses of 5, 10, and 20 µM.

### PLD Inhibits ROS, TNF-α, IL-1 β, and IL-6 Production Induced by LPS

**Figure 2:**
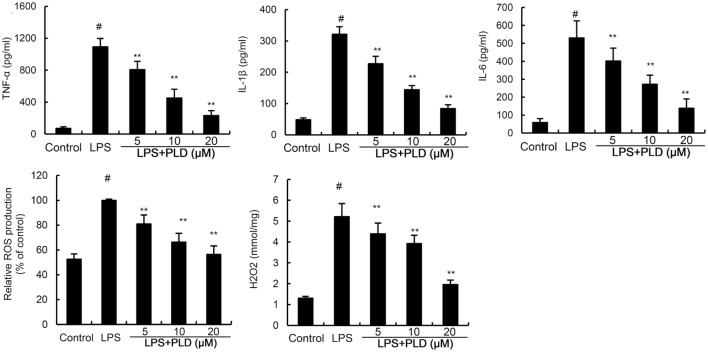
Effects of platycodin D (PLD) on LPS-induced ROS, TNF-α, IL-1β, and IL-6 production. Primary rat microglia cells were treated with PLD (5, 10, 20 µM) for 12 h and stimulated with LPS (0.5 µg/mL) for 24 h. The production of TNF-α, IL-1β, and IL-6 were measured by ELISA. The production of ROS was detected by DCFDA-cellular reactive oxygen species detection assay kit (Abcam, Cambridge, UK) and a colorimetric assay kit specific for H_2_O_2_ (Sigma, USA). The data presented are the means ± SD of three independent experiments and differences between mean values were assessed by one-way ANOVA with Tukey’s multiple comparison test. ^#^*p* < 0.05 vs. control group; *p < 0.05, **p < 0.01 vs. LPS group.

We detected the effects of PLD on inflammatory mediator production to assess the anti-inflammatory effects of PLD. The results showed that LPS significantly upregulated the levels of ROS, TNF-α, IL-1β, and IL-6 in primary rat microglia cells. However, treatment of PLD inhibited LPS-induced ROS, TNF-α, IL-1β, and IL-6 production in primary rat microglia cells (Figure 2).

### PLD Inhibits LPS-Induced NF-κB Activation

**Figure 3:**
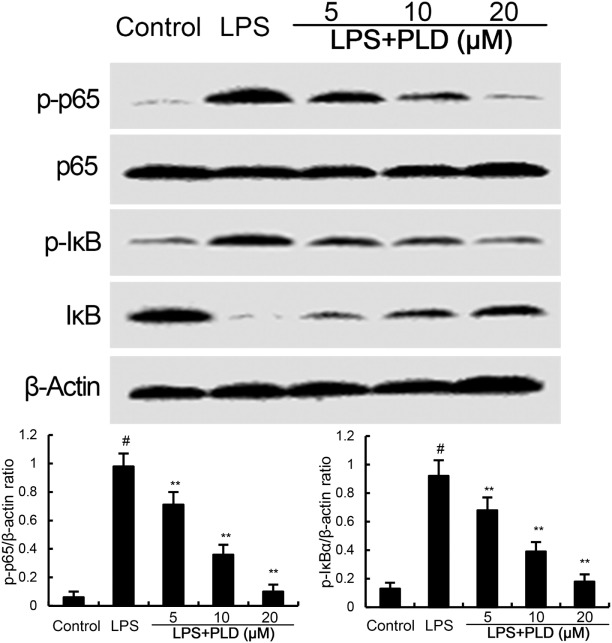
Platycodin D (PLD) inhibits LPS-induced NF-κB activation. Cells were treated with PLD (5, 10, 20 µM) for 12 h and stimulated with LPS (0.5 µg/mL) for 30 min. Protein samples were analyzed by western blotting. The antibodies used for western blotting were purchased from Cell Signaling Technology (Danvers, MA, USA). The values presented are the means ± SD of three independent experiments and differences between mean values were assessed by one-way ANOVA with Tukey’s multiple comparison test (^#^*p* < 0.05 vs. control group; *p < 0.05, **p < 0.01 vs. LPS group).

NF-κB, an important transcriptional factor, plays an important role in the regulation of inflammatory mediators. To clarify the mechanism of PLD, the effects of PLD on NF-κB activation were tested by western blot analysis. Treatment of PLD significantly inhibited LPS-induced NF-κB P65 and IκBα phosphorylation (Figure 3).

### PLD Inhibits LPS-Induced TLR4 Translocation into Lipid Rafts

**Figure 4:**
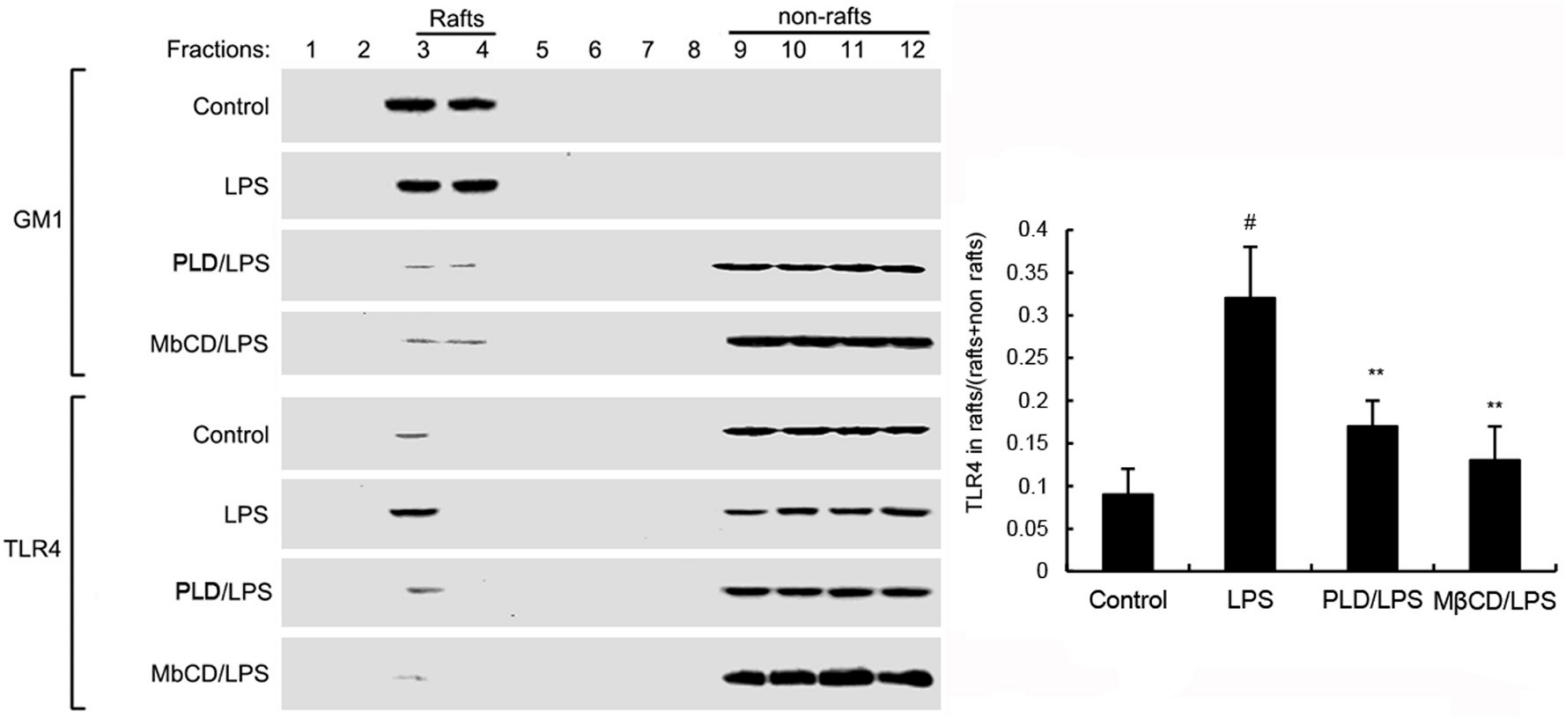
The recruitment of TLR4 to lipid rafts was inhibited by platycodin D (PLD). Cells were pretreated with PLD or MβCD, followed by treatment with LPS. The cells were lysed and subjected to discontinuous sucrose density gradient centrifugation as described in Section “Materials and Methods.” The fractions were analyzed by using CTxB conjugated to horseradish peroxidase (GM1) or anti-TLR4 primary antibody by western blotting. Fractions 3–4 correspond to lipid rafts. Representative blots of three separate experiments are shown. TLR4 content of macrophage lipid rafts was calculated as a percentage of total membrane TLR4 (lipid rafts + nonrafts). The values presented are the means ± SD of three independent experiments and differences between mean values were assessed by one-way ANOVA with Tukey multiple comparison test (^#^*p* < 0.05 vs. control group; *p < 0.05, **p < 0.01 vs. LPS group).

Activation of TLR4 leads to the activation of NF-κB. To investigate the mechanism of PLD, the effects of PLD on LPS-induced TLR4 translocation into lipid rafts were detected. GM1 is a marker for lipid raft. In the present study, we detected GM1 to identify lipid rafts. LPS stimulation induces translocation of TLR4 into lipid rafts. However, PLD significantly inhibited LPS-induced TLR4 translocation into lipid rafts (Figure 4).

### PLD Disrupts Lipid Rafts by Depleting Cholesterol

**Figure 5:**
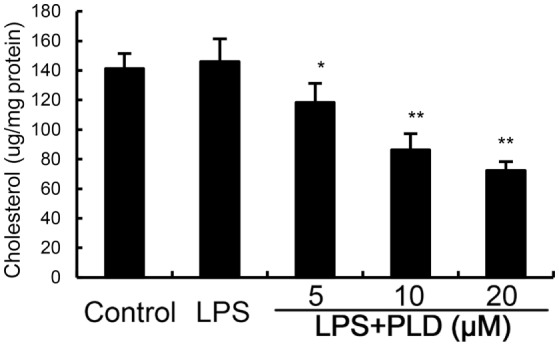
Effects of platycodin D (PLD) on lipid rafts cholesterol levels. Cells were treated with PLD (5, 10, 20 µM) for 12 h. Membrane cholesterol levels were measured by gas–liquid chromatography and the results were plotted as µg cholesterol/mg protein. The values presented are the means ± SD of three independent experiments and differences between mean values were assessed by one-way ANOVA with Tukey’s multiple comparison test (^#^*p* < 0.05 vs. control group; *p < 0.05, **p < 0.01 vs. LPS group).

Cholesterol is the main component of lipid rafts. To investigate the effects of PLD on the integrity of lipid rafts, we detected the effects of PLD on cholesterol level in lipid rafts. Treatment of PLD significantly decreased the level of cholesterol in lipid rafts which results in the disrupting of lipid rafts (Figure 5). These results suggested that PLD disrupted lipid rafts by depleting cholesterol.

### Cholesterol Replenishment Prevents the Anti-inflammatory Effects of PLD

**Figure 6:**
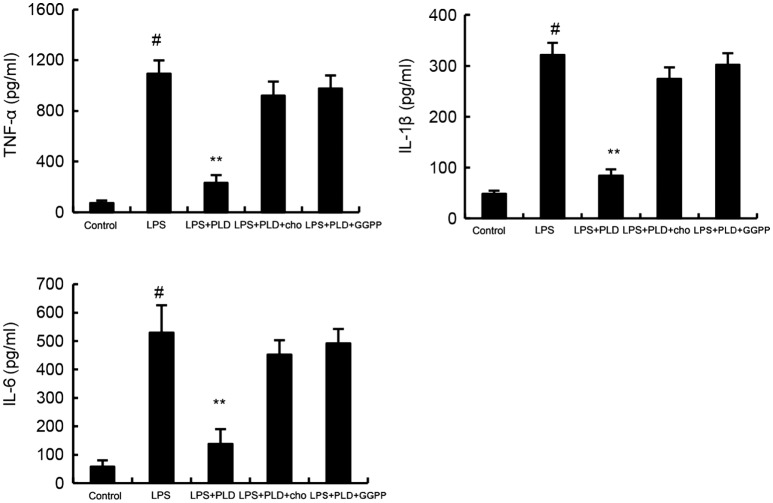
Cholesterol replenishment prevents the anti-inflammatory effect of platycodin D (PLD). Cells were treated with culture medium alone or medium containing PLD (5, 10, 20 µM) or MβCD (10 mM) at 37°C for 60 min. Subsequently the cells were washed with PBS and incubated with medium alone or medium containing water-soluble cholesterol (84 µg/ml) for 30 min. Cells were treated with LPS for 24 h. Levels of TNF-α, IL-1β, and IL-6 in culture supernatants were measured by ELISA. Effects of LXRα inhibitor geranylgeranyl pyrophosphate (GGPP) on the anti-inflammatory effects of PLD. Cells were treated with GGPP for 2 h. Then, the cells were treated with PLD for 12 h and stimulated by LPS. The productions of inflammatory cytokines were detected. The values presented are the means ± SD of three independent experiments and differences between mean values were assessed by one-way ANOVA with Tukey’s multiple comparison test (^#^*p* < 0.05 vs. control group; *p < 0.05, **p < 0.01 vs. LPS group).

To confirm whether cholesterol was involved in the anti-inflammatory mechanism of PLD, we used cholesterol replenishment experiments to confirm it. The results showed that when cholesterol was added, the anti-inflammatory effects of PLD were abolished (Figure 6).

### Effects of PLD on LXRα–ABCA1 Signaling Pathway

**Figure 7:**
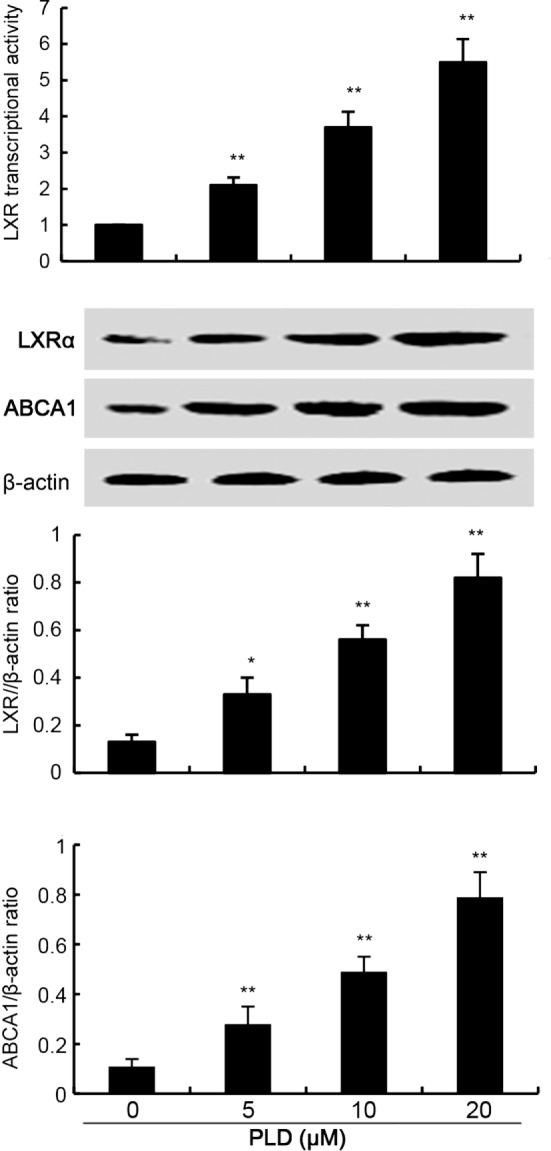
Effects of platycodin D (PLD) on LXR transcriptional activity and LXRα, ABCG1 expression. Cells were transfected with LXRE-driven luciferase reporter vector (LXRE-tk-Luc) and β-galactosidase control vector (Promega). Six hours later, cells were treated with PLD for 12 h. Relative luciferase activity was determined by normalization with β-galactosidase activity (*p < 0.05, **p < 0.01). Effect of PLD on LXRα and ABCG1 expression. Cells were treated with PLD (5, 10, 20 µM) for 12 h. Protein samples were analyzed by western blot with specific antibodies. β-Actin was used as a control. The values presented are the means ± SD of three independent experiments and differences between mean values were assessed by one-way ANOVA with Tukey’s multiple comparison test (*p < 0.05, **p < 0.01).

LXRα–ABCA1 signaling pathway is involved in the regulation of cholesterol efflux. To investigate the mechanism that PLD decreased the level of cholesterol, the effects of PLD on LXRα–ABCA1 signaling pathway were detected. In this study, PLD significantly upregulated the transcriptional activity of LXRα by luciferase reporter gene assay (Figure 7). Furthermore, PLD was found to upregulate the expression of LXRα and ABCA1.

### The Anti-inflammatory Effects of PLD Is LXRα Dependent

**Figure 8:**
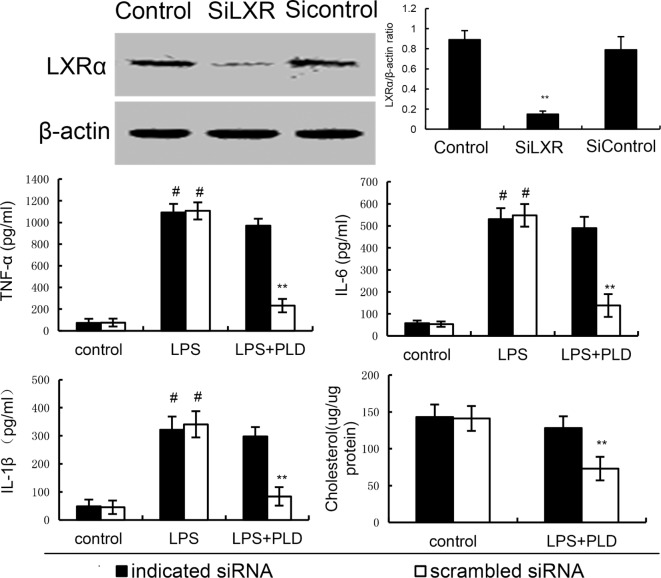
Knockdown of LXRα abrogated the effects of platycodin D (PLD) on lipid rafts cholesterol levels, and LPS induces inflammatory response in primary rat microglia cells. Cells were transfected with a siRNA specific for LXRα, or a scrambledsiRNA (negative control) as indicated. Then, the cells were treated with PLD (20 µM) for 12 h. The effect of siRNA on LXRα expression was detected by western blotting. Lipid raft cholesterol levels were detected. Meanwhile, the cells were treated with PLD (20 µM) for 12 h and stimulated by LPS for 24 h. Levels of TNF-α, IL-1β, and IL-6 in culture supernatants were measured by ELISA. The data presented are the means ± SD of three independent experiments and differences between mean values were assessed by one-way ANOVA with Tukey’s multiple comparison test (^#^*p* < 0.05 vs. control group; *p < 0.05, **p < 0.01 vs. LPS group).

To further confirm the anti-inflammatory mechanism of PLD, LXRα was knockdown by specific siRNA. The results showed that once LXRα was knockdown, the effects of PLD on cholesterol levels, the expression of cytokines TNF-α, IL-1β, and IL-6 induced by LPS were reversed (Figure 8). Furthermore, our results showed that the inhibition of PLD on TNF-α, IL-1β, and IL-6 production were reversed by LXRα antagonist geranylgeranyl pyrophosphate (Figure 6). Taken together, PLD exhibited anti-inflammatory effects by activating LXRα.

## Discussion

Previous studies suggested that inhibition of microglia activation was useful in the treatment of neurodegenerative diseases (19). We found PLD inhibited microglia activation by suppressing ROS and inflammatory cytokines production. The anti-inflammatory mechanism of PLD was through activating LXRα–ABCA1 signaling pathway and inhibiting TLR4 translocation into lipid rafts, thereby inhibiting LPS-induced inflammatory responses.

Microglia, the major immune cells in the central nervous system, plays an important role in host innate immune response (20). LPS induced the production of ROS and inflammatory cytokines, such as TNF-α, IL-1β, and IL-6 (21). These cytokines play critical roles in the pathogenesis of neurodegenerative diseases. In this study, PLD significantly inhibited microglia activation by suppressing ROS, TNF-α, IL-1β, and IL-6 production. NF-κB plays a critical role in the regulation of inflammatory cytokines production (22, 23). Our results showed that PLD inhibited LPS-induced inflammatory cytokines production by inhibiting NF-κB activation. Our results were consistent with previous studies (12, 24, 25). They suggested that PLD inhibited LPS-induced inflammation by inhibiting NF-κB activation.

TLR4 is the major receptor of LPS (26). Activating of TLR4 signaling pathway leads to NF-κB activation, which subsequently induces the production of inflammatory cytokines production (27). Lipid rafts are membrane domains that are rich in cholesterol and sphingolipids (28). Previous studies showed that lipid rafts played an important role in TLR4 signaling pathway (29). LPS-mediated TLR4 trafficking to lipid rafts represents an early event in signal initiation of immune cells (30). Studies showed that inhibition of TLR4 trafficking to lipid rafts could inhibit LPS-induced inflammatory responses (31). In this study, our results showed that PLD significantly inhibited LPS-induced TLR4 trafficking to lipid rafts. Furthermore, the effects of PLD on the level of cholesterol in lipid rafts were detected in this study. Our results showed that PLD disrupted the formation of lipid rafts by decreasing the level of cholesterol.

LXRα is a ligand-activated transcription factor that belongs to the nuclear receptor superfamily (32). LXRα is an important regulator of intracellular cholesterol (33). Activating of LXRα induces the expression of ABCA1 (34). ABCA1 is a lipid pump that effluxes cholesterol and phospholipid out of cells (35). In this study, we detected the effects of PLD on LXRα–ABCA1 signaling pathway. The results showed that PLD could activate LXRα and upregulated the expression of LXRα and ABCA1. PLD decreased the level of cholesterol by activating LXRα–ABCA1 signaling pathway. To further confirm the mechanism of PLD, LXRα was knockdown by siRNA. Once LXRα was knockdown, the anti-inflammatory effects of PLD were reversed. PLD exhibited its anti-inflammatory effects by activating LXRα.

In conclusion, the results of this study showed that PLD inhibited LPS-induced inflammation in microglia cells by activating LXRα–ABCA1 signaling pathway, which subsequently disrupting lipid rafts and inhibiting TLR4 translocation into lipid rafts, thereby inhibiting LPS-induced inflammatory responses. Previous studies showed that LXRα agonist could enhance blood–brain barrier integrity and attenuate blood–brain barrier disruption (36, 37). Furthermore, previous studies showed that Saikosaponin a, glycyrrhizin, and ginsenoside could attenuate neuroinflammation in the brain (38–40). Therefore, we speculated these compounds had the ability to penetrate blood–brain barrier. PLD has the similar chemical structure with these compounds and all these compounds could activate LXRα. Therefore, we speculated PLD might have the ability to penetrate blood–brain barrier. And further studies need to confirm this and detect the protective effects of PLD on neurodegenerative diseases.

## Author Contributions

YF, BL, and FL designed the project and experiments. YF, ZX, BL, JJW, and JXW carried out most of the experiments. YF and FL wrote the manuscript. YF, BL, and FL carried out statistical analysis and prepared figures. All authors reviewed the manuscript.

## Conflict of Interest Statement

The authors declare that the research was conducted in the absence of any commercial or financial relationships that could be construed as a potential conflict of interest.
